# Two Decades With Chronic Actinic Granuloma: A Case Report

**DOI:** 10.7759/cureus.91979

**Published:** 2025-09-10

**Authors:** Neena Edupuganti, Nathan Sagasser, Melinda Greenfield

**Affiliations:** 1 Osteopathic Medicine, Philadelphia College of Osteopathic Medicine, Suwanee, USA; 2 Osteopathic Medicine, Philadelphia College of Osteopathic Medicine, Moultrie, USA; 3 Dermatology, Advanced Dermatology and Cosmetic Surgery, Jacksonville, USA

**Keywords:** actinic granuloma, annular plaques, elastophagocytosis, idiopathic granulomatous dermatosis, o’brien’s granuloma

## Abstract

Actinic granuloma (AG), also known as O’Brien’s granuloma, is a rare idiopathic granulomatous dermatosis affecting sun-exposed areas, predominantly in older individuals. It presents as erythematous papules that expand into annular plaques with central atrophy and serpiginous borders. The pathogenesis of AG remains unclear, but it is hypothesized to involve an autoimmune response to actinically damaged elastic fibers. Diagnosis is confirmed through histopathological examination, and treatment options vary widely with inconsistent outcomes. Here, we present a case of an individual with a 20-year history of AG that was refractory to treatment with topical steroids and topical antifungals. This case highlights the need for heightened awareness surrounding this condition in order to help patients better manage this disease.

## Introduction

Actinic granuloma (AG), also known as O’Brien’s granuloma, is a rare, idiopathic granulomatous dermatosis that presents in sun-exposed areas of the skin in older individuals [1-3]. AG initially presents as small papules that progressively expand into annular plaques with atrophic or hypopigmented centers and elevated serpiginous borders [1-3]. The pathogenesis of AG remains unknown; however, authors postulate that it is an inflammatory or autoimmune response to actinically damaged elastic fibers [1-3]. Histological examination is necessary in order to definitively diagnose AG and differentiate it from its mimics, such as granuloma annulare (GA) [1-3]. While there is no definitive treatment for AG, there are several reports of various therapeutic options with mixed results [4-12]. Here, we present the case of a 59-year-old man with a 20-year history of AG who was treated with topical steroids and topical antifungals and showed no improvement.

## Case presentation

**Figure 1:**
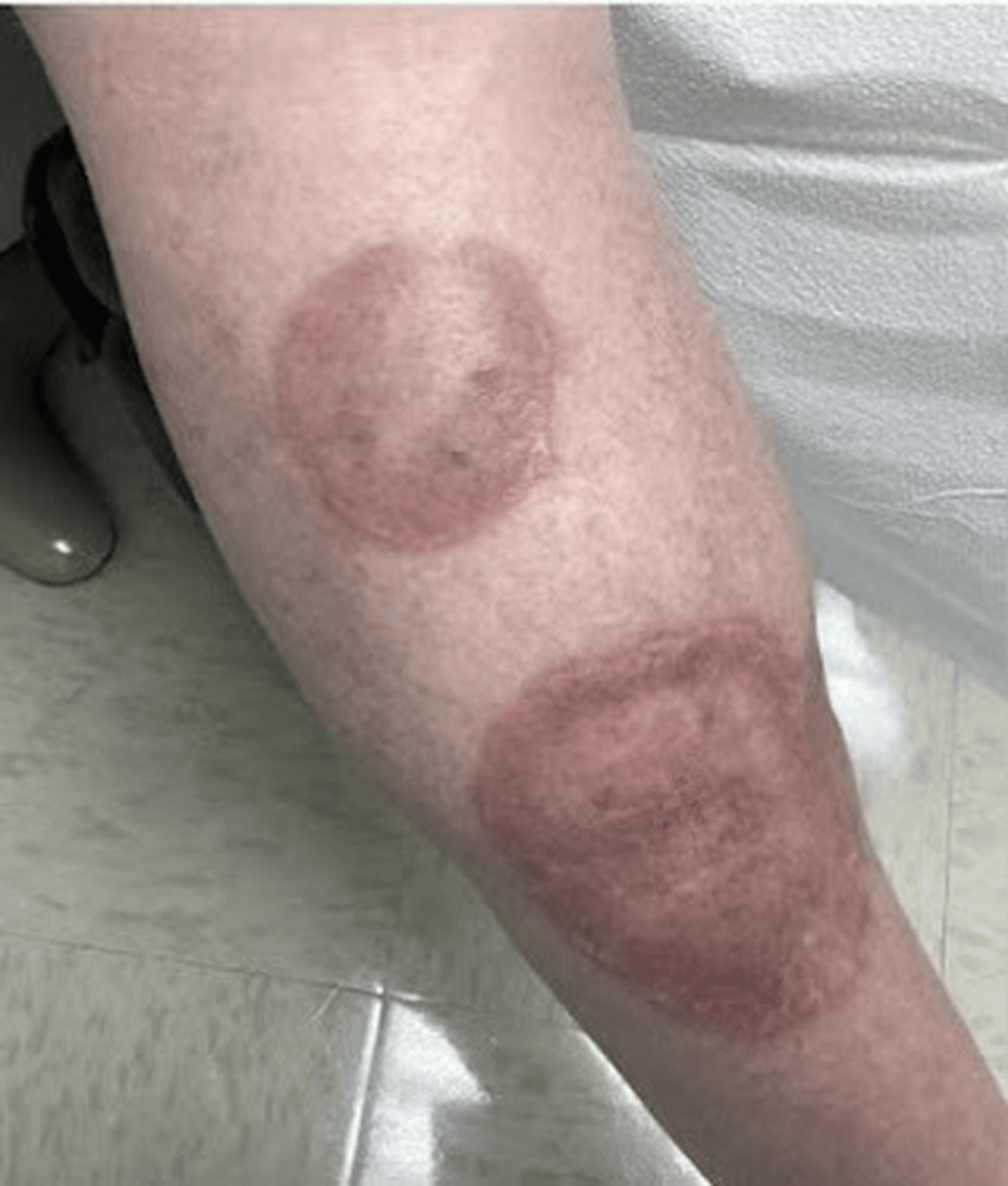
Erythematous, well-circumscribed plaques with raised, serpiginous borders and central atrophy and hypopigmentation located on the right distal pretibial region

**Figure 2:**
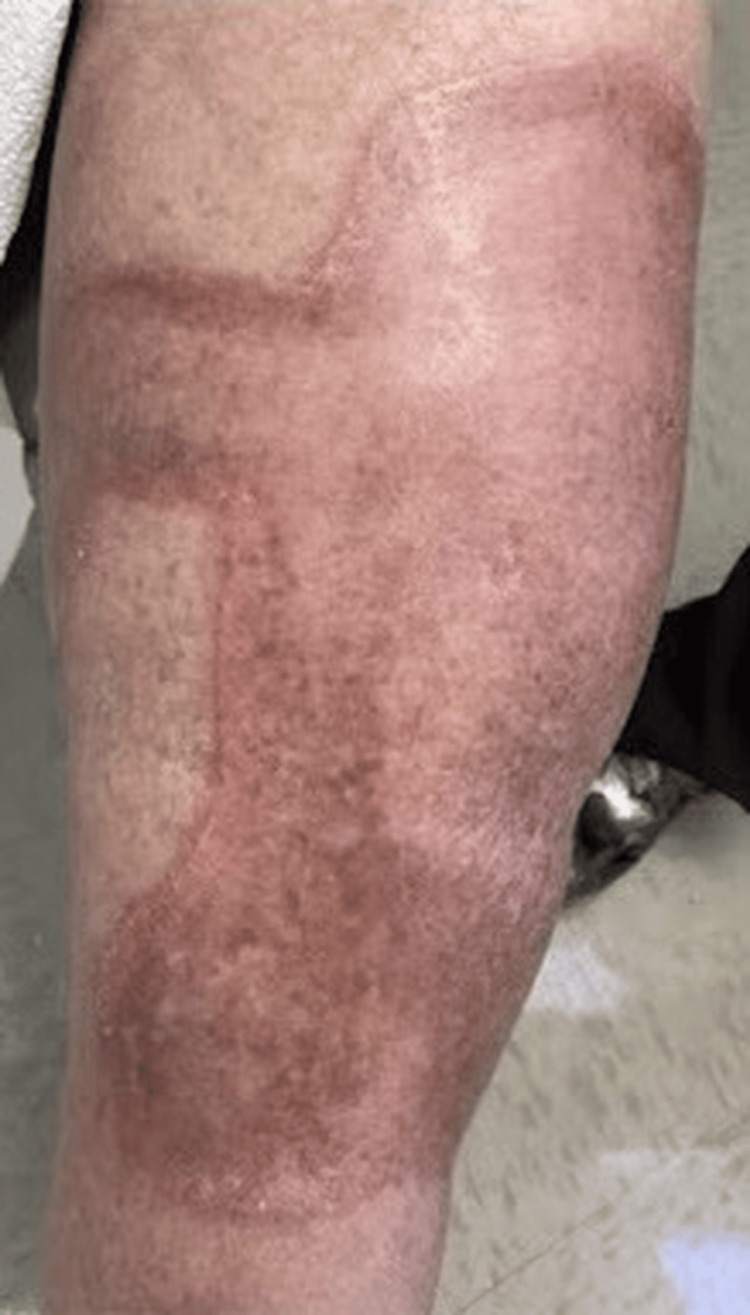
Erythematous, well-circumscribed plaque with raised, serpiginous borders and central atrophy and hypopigmentation located on the left distal pretibial region

A 59-year-old Caucasian male patient with no significant medical history presented to our outpatient dermatology clinic with longstanding skin lesions on his lower legs. On examination, the lesions presented as multiple erythematous, well-defined annular plaques with central atrophy and serpiginous borders, located bilaterally on the pretibial region (Figures 1, 2). The patient reported that the plaques have progressively increased in size but noted no associated symptoms, such as tenderness, pruritus, bleeding, or burning.

The patient had previously consulted two dermatologists, both of whom were unable to reach a definitive diagnosis. Previous treatments included topical corticosteroids and antifungal agents, neither of which yielded improvement. Notably, as a home inspector, he experiences a considerable daily exposure to ultraviolet radiation.

**Figure 3:**
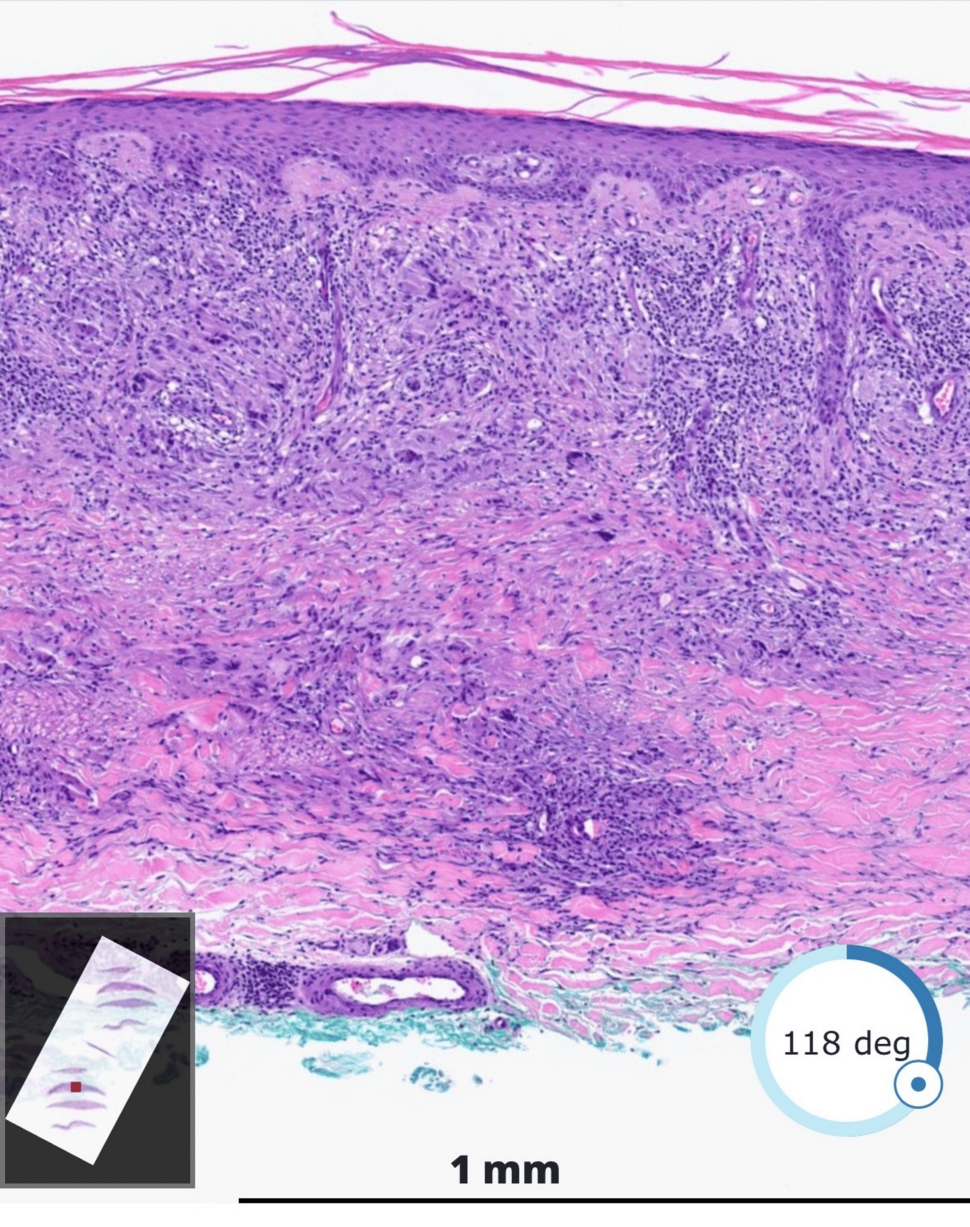
Shave biopsy (hematoxylin and eosin stain, magnification 2×) showing a dermal infiltrate of histiocytes and giant cells with damaged elastic fibers engulfed by giant cells

A shave biopsy performed on the left distal pretibial region revealed a dermal infiltrate of histiocytes and giant cells. Elastic fibers were present and appeared damaged. Some of the elastic fibers were engulfed by giant cells. Dermal mucin was not increased (Figure 3). A diagnosis of AG was confirmed. The patient was prescribed clobetasol propionate cream twice daily with no improvement. The patient was lost to follow-up, and therefore, additional treatment options could not be considered.

## Discussion

The differential diagnoses that were considered upon examination included granuloma annulare (GA) and necrobiosis lipoidica (NL). As these conditions may appear similar clinically, histopathological examination is needed to confirm the diagnosis. AG reveals a granulomatous reaction composed of multinucleated foreign body giant cells with elastophagocytosis and the absence of mucin [1,2,12]. In contrast, GA lesions are characterized by foci of necrobiotic collagen surrounded by palisading granulomas and can be distinguished from AG by the absence of multinucleated giant cells, elastophagocytosis, and the presence of mucin [1,2,12]. Similarly, NL will present similarly to GA with paslidaing granuloma, areas of necrobiosis, and the absence of multinucleated giant cells, distinguishing itself from AG [1,3,13]. 

While there is no definitive treatment for this condition, limiting sun exposure, regular sunscreen use, and topical, oral, and intralesional steroids are recommended [6]. In refractory cases, reports of acitretin, isotretinoin, cyclosporine, chloroquine, pentoxifylline, cryotherapy, psoralen ultraviolet A photochemotherapy, and methotrexate have been used with varying success [4-12]. In some cases, patients may experience complete resolution of the lesions without any treatment intervention [11].

Given the chronic nature of AG, it is crucial to educate patients on the importance of adherence to long-term management of this condition, which includes sun protection and regular dermatologic follow-up to prevent the development of new lesions and monitor the progression of existing lesions, respectively. 

This case highlights the importance of heightened awareness and recognition of this condition in the differential diagnosis of patients who present with single or multiple annular plaques. AG is often misdiagnosed due to its variable clinical morphology, histopathologic overlap with other granulomatous diseases, and lack of standardized diagnostic criteria in the dermatopathology literature. It is important that cases are continued to be published in order for us to better be able to help patients manage this condition, as it is an aesthetically disfiguring condition and can greatly affect the patient's quality of life.

## Conclusions

AG remains a rare and often underrecognized granulomatous dermatosis that can be clinically misdiagnosed without histopathologic confirmation. This case underscores the importance of considering AG in the differential diagnosis of annular plaques, particularly in patients with significant sun exposure and longstanding, treatment-resistant lesions. Histopathology is important in preventing misdiagnosis of AG from other granulomatous dermatoses with overlapping clinical features, such as GA, NL, and neoplastic or infectious processes. AG can be distinguished by granulomatous inflammation with multinucleated giant cells, prominent elastolysis, and elastophagocytosis, typically in the absence of mucin and necrobiosis. Despite limited response to conventional therapies, accurate diagnosis allows for more informed management strategies. Ongoing documentation and reporting of such cases are essential to improving recognition, guiding treatment, and ultimately enhancing patient outcomes.

## References

[1] O'Brien JP (1975). Actinic granuloma. An annular connective tissue disorder affecting sun- and heat-damaged (elastotic) skin. Arch Dermatol.

[2] Al-Hoqail IA, Al-Ghamdi AM, Martinka M, Crawford RI (2002). Actinic granuloma is a unique and distinct entity: a comparative study with granuloma annulare. Am J Dermatopathol.

[3] Gutiérrez-González E, Pereiro M Jr, Toribio J (2015). Elastolytic actinic giant cell granuloma. Dermatol Clin.

[4] Thacker PM, Nayak K, Lobo FD, Govindasamy P (2016). O'Brien's granuloma-a case report. J Clin Diagn Res.

[5] Jeha GM, Luckett KO, Kole L (2020). Actinic granuloma responding to doxycycline. JAAD Case Rep.

[6] Stein JA, Fangman B, Strober B (2007). Actinic granuloma. Dermatol Online J.

[7] Mamalis A, Ho D, Parsi KK, Jagdeo J (2018). Successful treatment of actinic granuloma with pulsed-dye laser and fractionated carbon dioxide laser. Dermatol Surg.

[8] Stefanaki C, Panagiotopoulos A, Kostakis P, Stefanaki K, Petridis A (2005). Actinic granuloma successfully treated with acitretin. Int J Dermatol.

[9] Rubio FA, Robayna G, Pizarro A, de Lucas R, Herranz P, Casado M (1998). Actinic granuloma and vitiligo treated with pentoxifylline. Int J Dermatol.

[10] Ratnavel RC, Grant JW, Handfield-Jones SE, Norris PG (1995). O'Brien's actinic granuloma: response to isotretinoin. J R Soc Med.

[11] Lazzarini R, Rotter A, Farias DC, Muller H (2011). O'Brien's actinic granuloma: an unusually extensive presentation. An Bras Dermatol.

[12] Michaelis TC, Woodcock JL, Basic KK (2017). Actinic granuloma presenting as tender, linear plaques on the lateral fingers in a patient with newly diagnosed esophageal cancer. JAAD Case Rep.

[13] Hanke CW, Bailin PL, Roenigk HH Jr (1979). Annular elastolytic giant cell granuloma. A clinicopathologic study of five cases and a review of similar entities. J Am Acad Dermatol.

